# A Prognostic Cuproptosis-Related LncRNA Signature for Colon Adenocarcinoma

**DOI:** 10.1155/2023/5925935

**Published:** 2023-02-17

**Authors:** Like Zhong, Junfeng Zhu, Qi Shu, Gaoqi Xu, Chaoneng He, Luo Fang

**Affiliations:** ^1^The Department of Pharmacy, Zhejiang Cancer Hospital, Hangzhou, China; ^2^Key Laboratory of Prevention, Diagnosis and Therapy of Upper Gastrointestinal Cancer of Zhejiang Province, Hangzhou, China

## Abstract

**Background:**

Cuproptosis, a recently discovered form of cell death, is caused by copper levels exceeding homeostasis thresholds. Although Cu has a potential role in colon adenocarcinoma (COAD), its role in the development of COAD remains unclear.

**Methods:**

In this study, 426 patients with COAD were extracted from the Cancer Genome Atlas (TCGA) database. The Pearson correlation algorithm was used to identify cuproptosis-related lncRNAs. Using the univariate Cox regression analysis, the least absolute shrinkage and selection operator (LASSO) was used to select cuproptosis-related lncRNAs associated with COAD overall survival (OS). A risk model was established based on the multivariate Cox regression analysis. A nomogram model was used to evaluate the prognostic signature based on the risk model. Finally, mutational burden and sensitivity analyses of chemotherapy drugs were performed for COAD patients in the low- and high-risk groups.

**Result:**

Ten cuproptosis-related lncRNAs were identified and a novel risk model was constructed. A signature based on ten cuproptosis-related lncRNAs was an independent prognostic predictor for COAD. Mutational burden analysis suggested that patients with high-risk scores had higher mutation frequency and shorter survival.

**Conclusion:**

Constructing a risk model based on the ten cuproptosis-related lncRNAs could accurately predict the prognosis of COAD patients, providing a fresh perspective for future research on COAD.

## 1. Introduction

Colonic adenocarcinoma (COAD) is the most common histological subtype of colorectal cancer and is one of the leading causes of cancer mortality [1]. With the development of substantive treatment strategies, including surgery, neoadjuvant therapy, and targeted therapy, the overall prognosis for patients with COAD has significantly improved [2]. At the same time, the importance of early diagnosis of COAD for prognosis is being increasingly recognized. The 5-year survival rate of patients with early diagnosis is approximately 90%, but only 10% for patients diagnosed with advanced metastatic disease [1]. Identifying novel biomarkers for tumor diagnosis and prognosis has been shown to benefit the treatment of diverse tumor types [3–6]. Therefore, there is still an urgent need to identify novel prognostic biomarkers associated with metastasis to facilitate the timely diagnosis and earlier application of appropriate, individualized therapy.

Long noncoding RNAs (lncRNAs) are transcripts over 200 nucleotides in length with no significant protein-coding function [7]. By modulating gene expression, lncRNAs have been reported to play important roles in many physiological processes and disease progression [8]. In COAD, a variety of lncRNAs have been reported to be highly expressed and have been associated with multiple tumor-related biological processes, including proliferation, chemical resistance, and epithelial-mesenchymal transformation [9–12]. These lncRNAs have been associated with the activation of multiple signaling pathways, including WNT, PI3K/Akt, and PPAR [13]. Considering the roles of these pathways in the occurrence and development of COAD [14, 15], lncRNAs are likely to be significant factors in tailoring individualized therapies. Several studies have identified lncRNAs as potential therapeutic targets [16–18]. Overexpression of LINC00152 has been shown to promote the expression of fascin actin-binding protein 1 (FSCN1) by binding mir-632 and mir-185-3p, leading to proliferation and metastasis [19]. As reviewed in 2022, lncRNAs including DCST1-AS1, LINC01569, KCNQ1OT1, and LINC00997 were considered to take an active part in carcinogenesis by influencing cell metastasis, drug resistance, radio-resistance, and tumor microenvironment interaction [20]. However, the role of lncRNAs in COAD has not been completely elucidated.

Cu levels are elevated in the serum and tissues of multiple solid tumors, including colorectal tumors [21]. However, its role is not fully understood. On one hand, in addition to acting as a cofactor for key metabolic enzymes, Cu also directly promotes tumor growth by acting as a cofactor for signaling molecules such as MEK1, which transduces carcinogenic BRAF signals to ERK1/2 [22], suggesting that it may have a key role in cancer progression. On the other hand, the ion carrier elesclomol mediates copper overload in colorectal cancer cells and induces copper-dependent cell death by degrading ATP7A [23].

This cell death pathway, caused by copper levels exceeding homeostasis thresholds, is called copper death or cuproptosis [24]. It relies on mitochondrial respiration [25]. Copper binds directly to the lipid components of the tricarboxylic acid (TCA) cycle, resulting in the accumulation of lipoacylated proteins, followed by the loss of iron-sulfur cluster proteins, resulting in proteotoxic stress and cell death [26]. Cuproptosis caused by copper overload has been shown to predict tumor prognosis and judge immune and drug responses in a variety of tumors, including head and neck squamous cell carcinoma, breast cancer, and cervical cancer [27–30]. However, there is no relevant report found in COAD. Therefore, the double-edged role of copper in colorectal cancer and its influence on prognosis need to be further analyzed and understood.

In this study, we examined cuproptosis-associated lncRNAs in the clinical context of COAD using the Cancer Genome Atlas (TCGA) database. We constructed a risk model to evaluate the prognostic ability of cuproptosis-associated lncRNAs in patients with COAD. The tumor mutational burden and sensitivity analysis of chemotherapy drugs were also assessed. Taken together, our findings provide new insights into potential therapeutic strategies for patients with COAD.

## 2. Materials and Methods

### 2.1. Data Collection

Gene expression matrices and clinical information for patients with COAD were obtained from the Cancer Genome Atlas database (https://portal.gdc.cancer.gov/). We identified 426 such samples for inclusion. The gene expression matrices were merged using a Perl script for further analysis. Genes encoding lncRNAs and mRNAs were annotated and classified using the Human Genome Browser, GRCh38.p13 (https://asia.ensembl.org/index.html). Survival time, survival status, age, sex, stage, and TNM stage were extracted from the TCGA database using Perl scripts. All data and clinical information used in this study were obtained from a public database; therefore, neither approval from the ethics committee nor written informed consent from patients was required.

### 2.2. Identification of Cuproptosis-Related lncRNAs

Expression data for cuproptosis-related genes were obtained from a previous study [26]. Expression data were extracted using Perl scripts, and Pearson's correlation algorithm was used to identify cuproptosis-related lncRNAs. With the threshold setting at |correlation coefficient| > 0.4, *P*value <0.001 (*r* > 0.4, *P* < 0.001), 870 lncRNAs were identified as cuproptosis-related lncRNAs for further analysis (Supplementary Table 1).

### 2.3. Prognostic Signature Construction

Based on univariate Cox regression analysis, the least absolute shrinkage and selection operator (LASSO) algorithm was performed using the R package “glmnet.” The multivariate Cox regression analysis was used to evaluate the lncRNA signature as an independent prognostic factor for patient survival. Risk scores for each patient were calculated using the following formula: risk scores = ∑_*i*=1_^*n*^Coef(*i*) × *x*(*i*), where Coef(*i*) represents the correlation regression coefficient and *x*(*i*) is the expression level of cuproptosis-related lncRNAs. Patients with COAD were divided into low- and high-risk groups based on median risk scores. Kaplan–Meier survival analysis was employed to assess the difference in OS rates in the low- and high-risk groups using the log-rank algorithm. A 3D principal component analysis (3D-PCA) was conducted to assess the difference in signatures between low- and high-risk patients using the R package “ggplot2.”

### 2.4. Consensus Clustering Analysis

According to the prognostic cuproptosis-related genes, consensus clustering was performed using the R package “ConsensusClusterPlus.” The clustering was established on the grounds of partitioning around medoids with “Euclidean” distances, and 1,000 verifications were performed. Finally, with the optimal classification of *K* = 2–9, the patients with COAD were clustered into two subtypes for further analysis.

### 2.5. Risk Model Independence

The univariate and multivariate Cox regression analyses were used to assess risk scores as independent prognostic factors for COAD. A subtype analysis was conducted to confirm the independence of the risk model. To further determine whether the risk score was independent of other clinical variables, including age, Gleason score, PSA value, and T stage, patients were regrouped into new subtypes based on different clinical characteristics. According to median risk scores, patients in each subtype were stratified into low- and high-risk groups.

### 2.6. Somatic Mutation Analysis

Data from the COAD samples were obtained from TCGA in “maf” format using Perl scripts. A waterfall diagram was constructed using the “Maftools” package in the R software.

### 2.7. Drug Sensitivity Analysis

Based on the Genomics of Drug Sensitivity Genomics in Cancer (GDSC), the drug treatment response of each patient with COAD was predicted using the R package “pRRophetic.” Differences in IC_50_ values between low- and high-risk groups were analyzed using the “ggplot2” R package.

### 2.8. Gene Set Enrichment Analysis (GSEA)

For the low- and high-risk groups, 1,000 permutations were used and screened using the largest and smallest gene set filters of 500 and 15 genes, respectively. *P* values less than 0.05 were considered to be significantly different.

### 2.9. Statistical Analysis

All analyses were performed using the R software (version 3.6.0) and Perl scripts. The Wilcoxon rank sum test was applied to separately conduct group comparisons with *P* values less than 0.05, which was considered to be statistically significant.

## 3. Results and Discussion

### 3.1. Identification of Cuproptosis-Related lncRNAs

**Figure 1:**
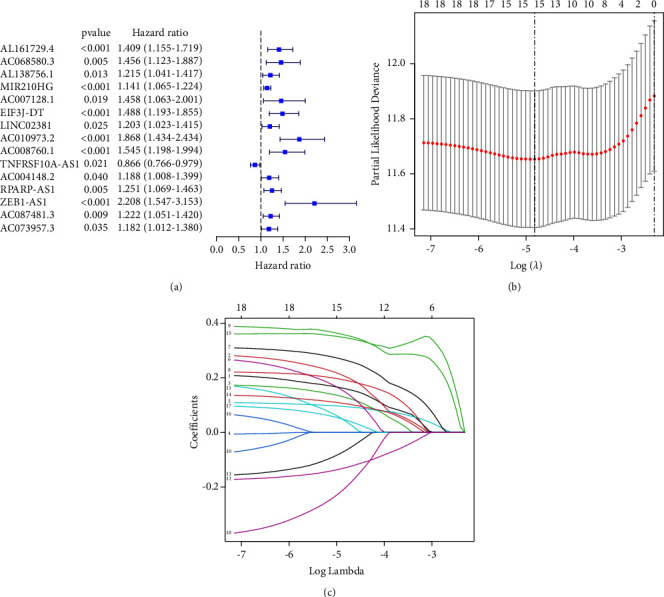
Identification and analysis of cuproptosis-related lncRNAs: (a) univariate Cox regression for 15 cuproptosis-related lncRNAs associations with COAD OS. (b-c) LASSO regression model showing coefficients and minimal lambda values.

A total of 14,142 lncRNAs were collected from the TGCA COAD RNA-Seq matrix. To identify lncRNAs related to cuproptosis, correlations between the expression of cuproptosis genes and lncRNAs were calculated, yielding a total of 870 candidate lncRNAs. Using the univariate Cox regression analysis, 15 cuproptosis-related lncRNAs associated with OS were selected using the least absolute shrinkage and selection operator (LASSO) algorithm (Figure 1, Supplementary Table 2).

### 3.2. Risk Model Construction

**Figure 2:**
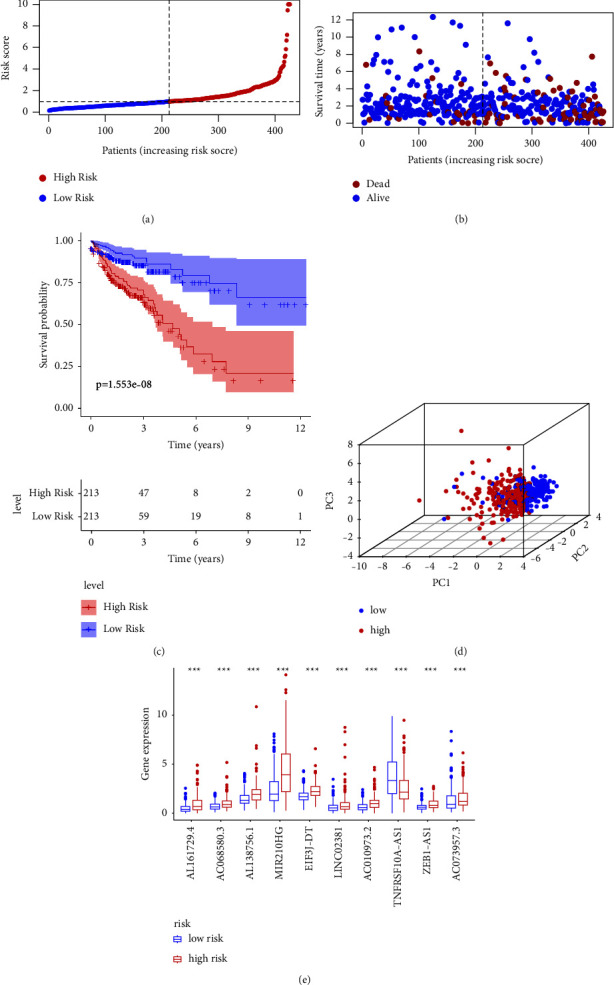
Risk model based on expression levels of ten cuproptosis-related lncRNAs: (a) distribution of risk scores; (b) scatter dot plot showing correlation of survival time and risk score; (c) Kaplan–Meier survival analysis; (d) principal component analysis (PCA) showing significant separation between low- and high-risk groups; (e) boxplot of expression levels of the ten selected lncRNAs.

From the multivariate Cox regression analysis, 10 cuproptosis-related lncRNAs were selected to construct a risk model. Risk scores for each patient were calculated using the following formula: risk scores = (0.24 × expression level of AL161729.4) + (0.35 × expression level of AC068580.3) + (0.19 × expression level of AL138756.1) + (0.1 × expression level of MIR210HG) + (0.38 × expression level of EIF3J-DT) + (0.17 × expression level of LINC02381) + (0.42 × expression level of AC010973.2) + (−0.15 × expression level of TNFRSF10A-AS1) + (0.42 × expression level of ZEB1-AS1) + (0.31 × expression level of AC073957.3). Using the median risk score, the COAD patients were divided into the following two groups: 213 patients in the low-risk group and 213 patients in the high-risk group. Patients were ranked according to the cuproptosis-related prognostic signature; the resulting scatter dot plot indicated that survival time was inversely correlated with risk score (Figures 2(a) and 2(b)). The Kaplan–Meier survival analysis showed that the OS of patients with high-risk scores was significantly shorter than that of those with low-risk scores (*P*=1.553*E* − 08, Figure 2(c)). A 3D principal component analysis (3D-PCA) produced a clear separation between low- and high-risk groups based on the selected lncRNAs (Figure 2(d)). Of the ten prognostic cuproptosis-related lncRNAs, AL161729.4, AC068580.3, AL138756.1, MIR210HG, EIF3J-DT, LINC02381, AC010973.2, ZEB1-AS1, and AC073957.3 were expressed at higher levels in the high-risk group, whereas TNFRSF10A-AS1 was expressed at higher levels in the low-risk group (Figure 2(e)). These results suggested that constructing a risk model based on the ten cuproptosis-related lncRNAs is prognostic for patients with COAD.

### 3.3. Training and Validation Cohorts

**Figure 3:**
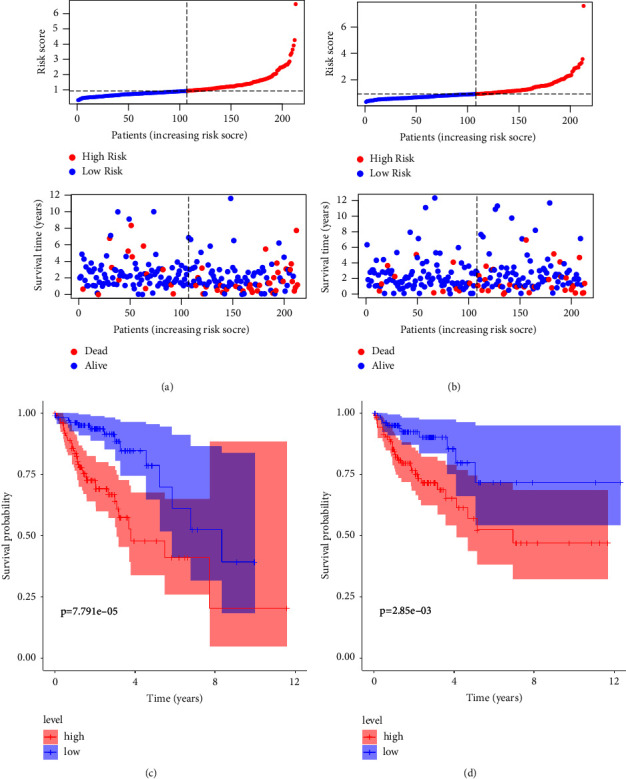
Testing of training and validation cohorts: (a-b) distribution of risk scores and scatter dot plots; (c-d) survival curves for training and validation cohorts.

The COAD patients were randomly classified into training and validation cohorts. In both cohorts, patients were ranked by median risk score. A scatter dot plot showed that survival times of COAD patients in the training and validation cohorts were conversely associated with risk scores (Figures 3(a) and 3(b)). The survival of patients with low-risk scores was higher than that of patients with high-risk scores in both cohorts (*P* < 0.001, Figures 3(c) and 3(d)). These results demonstrated that our risk model is accurate and reliable.

### 3.4. Independent Prognostic Analyses

**Figure 4:**
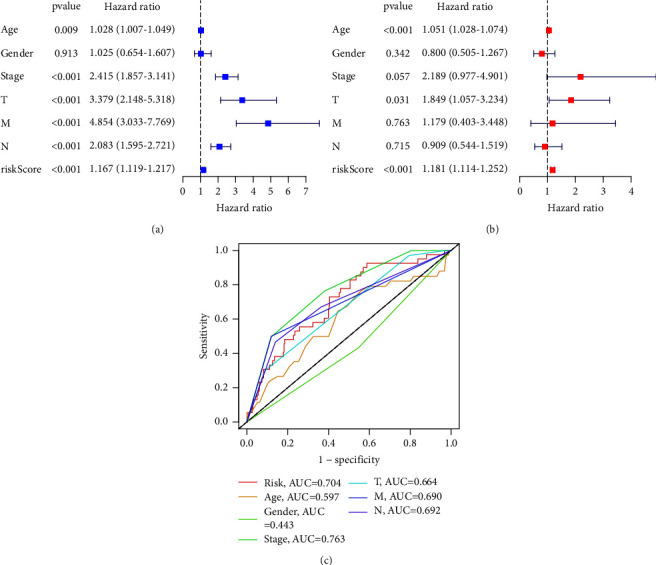
Independent prognostic analyses of the cuproptosis-related lncRNA signature: (a) univariate Cox regression showing the correlation between overall survival and clinicopathological characteristics; (b) multivariate Cox regression showing that age, T stage, and risk score are independent prognostic indicators for the overall survival; (c) receiver operating characteristic (ROC) curve analysis showing the prognostic accuracy for age, sex, stage, T stage, M stage, N stage, and risk score.

Univariate analysis indicated that age (hazard ratio (HR) = 1.028, *P*=0.009), stage (HR = 2.415, *P* < 0.001), T stage (HR = 3.379, *P* < 0.001), M stage (HR = 4.854, *P* < 0.001), N stage (HR = 2.083, *P* < 0.001), and the risk score (HR = 1.167, *P* < 0.001) were associated with OS (Figure 4(a)). Multivariate analysis indicated that age (HR = 1.051, *P* < 0.001), T stage (HR = 1.849, *P*=0.031), and risk score (HR = 1.181, *P* < 0.001) were significantly associated with OS in patients with COAD (Figure 4(b)). The AUC of the signature was 0.704 (Figure 4(c)). Taken together, these results indicate that prognostic signatures based on cuproptosis-related lncRNAs are independent prognostic factors in patients with COAD.

### 3.5. Correlations between lncRNA Risk Scores and Clinicopathological Characteristics

**Figure 5:**
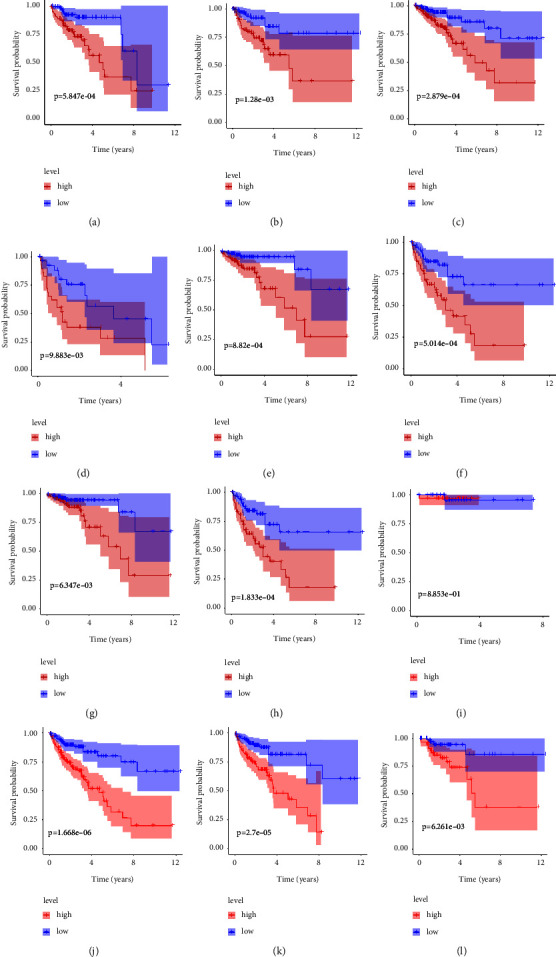
Kaplan–Meier analyses stratified by (a-b) sex, (c-d) M stage, (e-f) N stage, (g-h) S stage, (i-j) T stage, and (k-l) age.

Patients were classified by sex, M stage (M 0 vs. M 1), N stage (N 0 vs. N 1-2), S stage (S 1-2 vs. S 3-4), T stage (T 1-2 vs. T 3-4), and age (≥65 vs. <65). Kaplan–Meier analysis showed that survival of patients with low-risk scores was higher than that of patients with high-risk scores, based on the prognostic signature among females (*P*=5.847*e* − 04), males (*P*=1.28*e* − 03), M 0 (*P*=2.879*e* − 04), M 1(*P*=9.833*e* − 03), N 0 (*P*=8.82*e* − 04), N 1-2 (*P*=5.014*e* − 04), S 1-2 (*P*=6.347*e* − 04), S 3-4 (*P*=1.833*e* − 04), T 3-4 (*P*=1.668*e* − 06), ≥65 (*P*=2.7*e* − 05), and <65 (*P*=2.61*e* − 03). However, the survival rate was similar between T-stage groups (Figure 5). These results indicate that the prognostic signature based on cuproptosis-related lncRNAs accurately predicts prognosis relative to clinicopathological characteristics.

### 3.6. Consensus Clustering Analysis for Cuproptosis-Related lncRNAs associated with COAD

**Figure 6:**
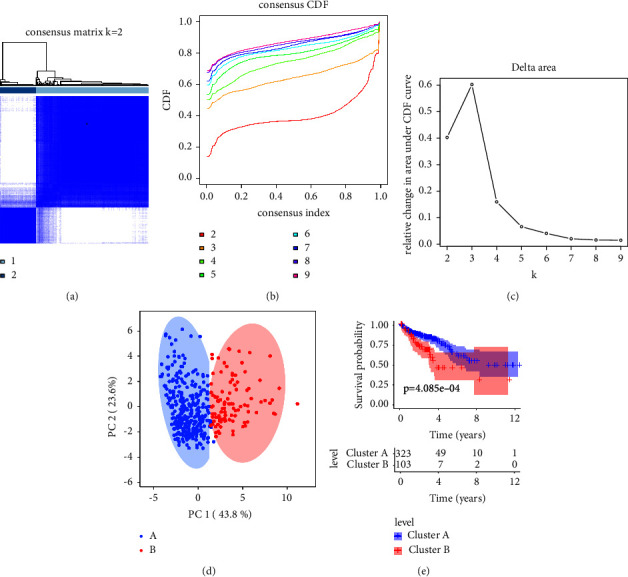
Consensus clustering analysis of patients with CM based on the ICD-related genes: (a) consensus clustering heatmap of the group at *k* = 2; (b) cumulative distribution function (CDF) curve for *k* = 2–9; (c) relative change in area under CDF curve for *k* = 2–9; (d) principal components analysis (PCA) shows a significant distribution pattern between cluster A and cluster B; (e) the Kaplan–Meier survival curve analysis reveals that the OS rate of patients in cluster A is higher than those in cluster B.

Thereafter, consensus clustering analysis was utilized to cluster the patients with COAD into different subgroups, and the result revealed an optimal classification for consensus clustering with *K* = 2 (Figures 6(a)–6(c)). Based on the prognostic cuproptosis-related lncRNAs, the patients with COAD were successfully divided into two subgroups, with 323 patients in Cluster A and 103 patients in Cluster B. The principal component analysis result illustrated a clear separation between Cluster A and Cluster B according to the prognostic cuproptosis-related lncRNAs (Figure 6(d)). The Kaplan–Meier survival curve analysis suggested that the patients in Cluster A had a higher OS rate than those in Cluster B (Figure 6(e)). These results demonstrate that the cuproptosis-related lncRNAs are associated with the prognosis of COAD.

### 3.7. Nomogram Construction

**Figure 7:**
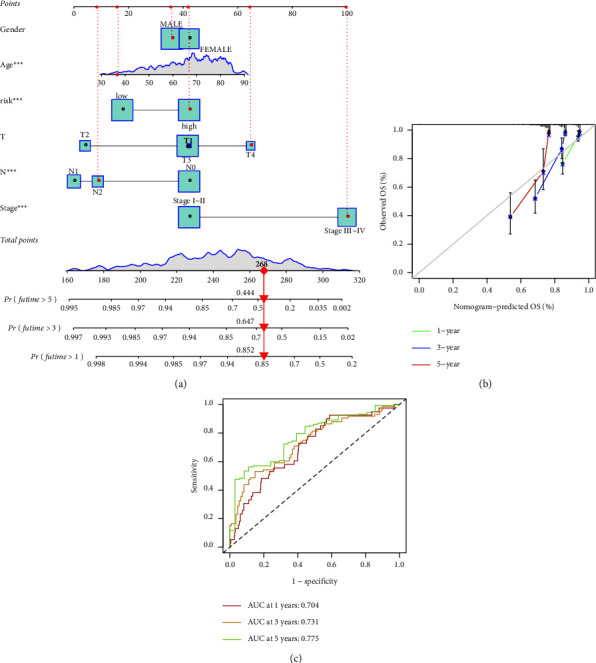
Prognostic nomogram: (a) nomogram using risk scores and clinical characteristics to predict 1-, 3-, and 5-year survival; (b) calibration curve to assess accuracy between predictive power and actual survival rates; (c) receiver operating characteristic (ROC) curve assessment of prognostic accuracy.

A nomogram was constructed to confirm the accuracy of the prognostic signature and clinicopathological characteristics (Figure 7(a)). It yielded a consistency index (C-index) of 0.727. Calibration curves indicated that the nomogram-predicted 1, 3, and 5-year survival rates were consistent with actual survival times (Figure 7(b)). Time-dependent ROC curves revealed that the AUCs of 1-, 3-, and 5-year were 0.704, 0.731, and 0.775, respectively, indicating satisfactory accuracy of the model (Figure 7(c)).

### 3.8. Tumor Mutational Burden (TMB) Analysis

**Figure 8:**
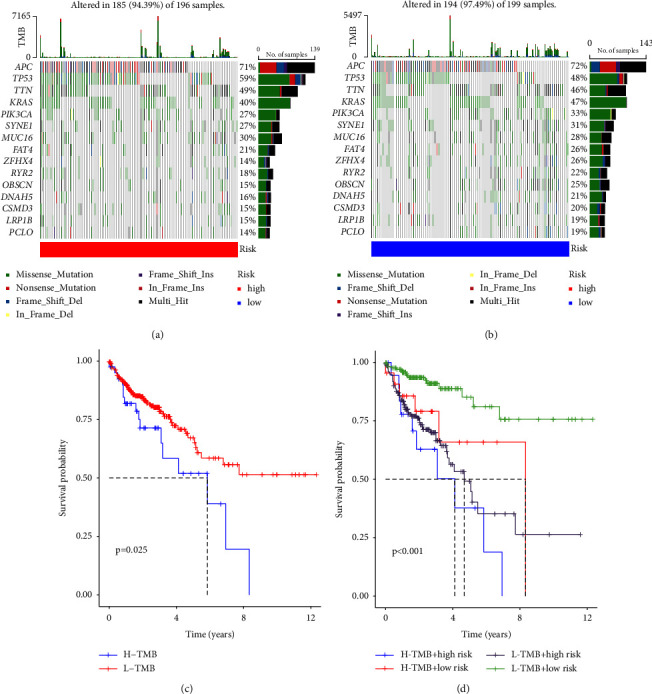
Tumor mutational burden analysis: (a) genes with the highest mutation rates in high-risk patients; (b) genes with the highest mutation rates in high-risk patients; (c) overall survival of patients with H-TMB and L-TMB; (d) overall survival of patients with L-TMB and H-TMB.

TMB indices for high-risk and low-risk genes were calculated. As shown in Figure 8(a), patients with high TMB had lower survival rates than those with low TMB (*P*=0.025). The mutation frequencies of high-risk genes were higher than those of low-risk genes. Survival of the high-TMB + high-risk panel was the lowest, followed by the low-TMB + high-risk, high-TMB + low-risk, and low-TMB + low-risk panels (Figure 8(b), *P* < 0.001). A waterfall diagram (Figure 8(c)) shows the top 30 mutation frequencies. In the low-risk group, mutations were detected in 194 out of 195 samples; APC (72%), TP53 (48%), TTN (46%), and KRAS (47%) had the highest mutation frequencies. In the high-risk group, mutations were detected in 185 out of 196 samples. The mutated genes with the highest frequency in the mutation map showed no significant difference compared with the previous group (Figure 8(d)).

### 3.9. Sensitivity to Chemotherapeutic Agents

**Figure 9:**
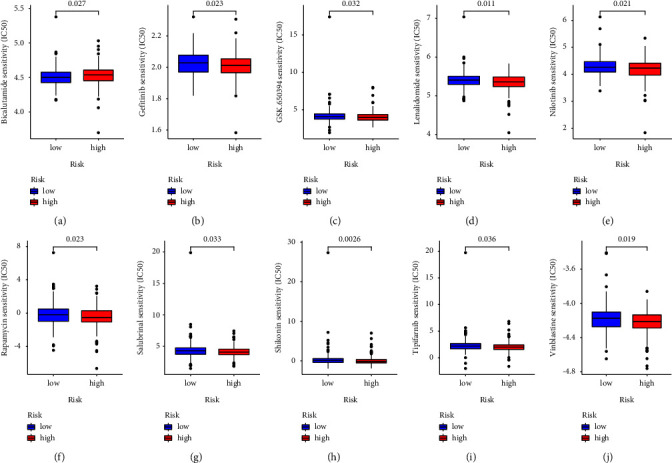
Drug sensitivities as a function of the risk group for (a) bicalutamide, (b) gefitinib, (c) GSK.650394, (d) lenalidomide, (e) nilotinib, (f) rapamycin, (g) salubrinal, (h) shikonin, (i) tipifarnib, and (j) vinblastine.

As chemotherapy is the primary treatment for newly diagnosed COAD, we compared IC_50_ values for several commonly used drugs between the low- and high-risk groups. IC_50_ values for high-risk COAD patients for nilotinib, rapamycin, gefitinib, salubrinal, GSK.650394, shikonin, lenalidomide, tipifarnib, and vinblastine were all lower (*P* < 0.05), while the IC_50_ for bicalutamide was higher in the high-risk group (Figure 9). These results provide preliminary evidence for clinical drug-use guidance.

### 3.10. Gene Set Enrichment Analysis (GSEA)

**Figure 10:**
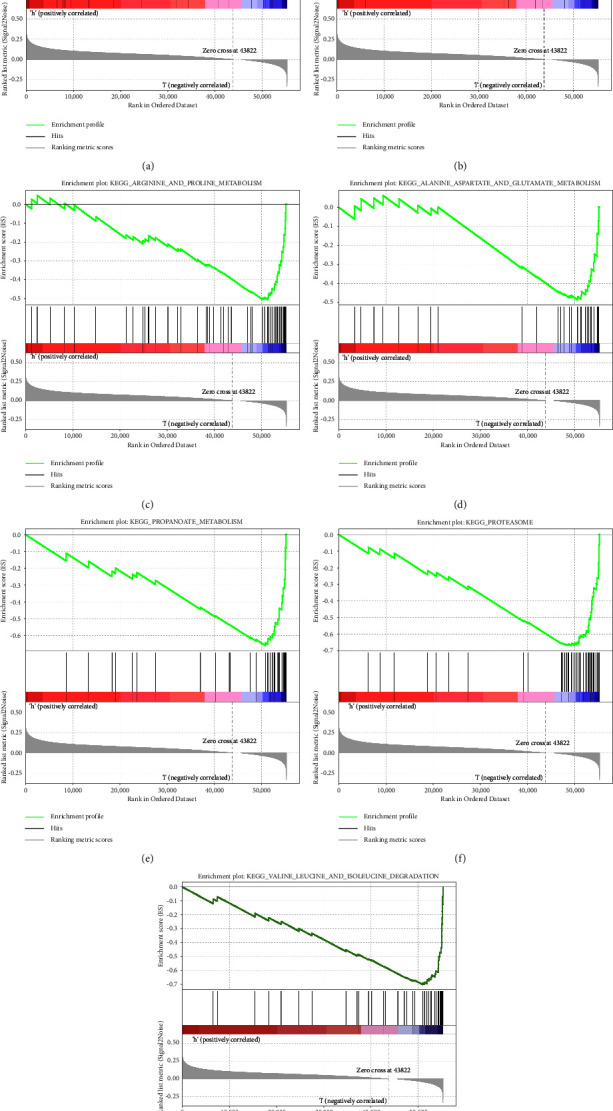
Gene set enrichment analyses: (a) mTOR signaling pathway; (b) citrate cycle TCCA cycle; (c) arginine and proline metabolism; (d) alanine, aspartate, and glutamate metabolism; (e) propanoate metabolism; (f) proteasome; (g) valine, leucine, and isoleucine degradation.

We found multiple KEGG signaling pathways that were dynamically enriched in the low-risk group compared to the high-risk group, including those involved in the citrate cycle of the TCA cycle; propanoate metabolism, arginine, and proline metabolism; alanine, aspartate, and glutamate metabolism; proteasome; and valine, leucine, and isoleucine degradation. Notably, the expression of components of the mTOR signaling pathway was significantly increased in the high-risk group (Figure 10). These results indicate that metabolic processes and cancer-related pathways may mediate the role of cuproptosis-related lncRNAs in patients with COAD.

## 4. Conclusions

Despite significant improvements in surgery, radiotherapy, chemotherapy, and immunotherapy, the 5-year COAD survival rate remains very low [1]. Therefore, it is important to identify potential biomarkers for diagnosis and treatment. In this study, we identified and validated a ten-gene feature that predicted survival in patients with COAD. This risk model may be clinically valuable for identifying patients for individualized, cuproptosis-inducing therapy.

Gene expression is regulated by the interaction of lncRNAs with RNA, DNA, and proteins through a variety of mechanisms, including regulation of transcription, mRNA stability, and translation [31]. In colon cancer, lncRNAs have been implicated in regulating cell proliferation, apoptosis, the cell cycle, cell migration and invasiveness, epithelial-mesenchymal transformation (EMT), cancer stem cells, and drug resistance [32]. Multiple types of lncRNAs have been correlated with COAD prognosis [33]. Copper-based therapies are considered to have great potential in cancer treatment; some are already in clinical trials. However, their anticancer potential has not been fully elucidated [34]. Cuproptosis is a newly discovered form of cell death that involves mitochondrial metabolic activity and has not been thoroughly studied in tumors [26]. In the current study, ten lncRNAs associated with cuproptosis were identified and included in a risk model. The Kaplan–Meier curve, time-dependent ROC curve, and Cox regression analysis all demonstrated the predictive ability of the risk model, indicating an independent predictor of COAD prognosis. Progressive preclinical and clinical evidence suggests that targeting mitochondrial metabolism has anticancer effects [35, 36]. Cuproptosis is associated with highly reactive mitochondrial oxidative phosphorylation (OXPHOS) [26]. Despite an increasing reliance on glycolysis, cells from many cancer types still exhibit functional OXPHOS [37]. In colon adenocarcinomas, stem cells have been reported to use mitochondrial OXPHOS to produce ATP and maintain mitochondrial function via the FOXM1/PRDX3 pathway, thereby maintaining their survival and stem-cell characteristics [38].

Among the lncRNAs screened, MIR210HG, EIF3J-DT, and ZEB1-AS1 have been extensively studied in tumors. MIR210HG promotes breast cancer progression through m6A modification mediated by IGF2BP1 [39]. IGF2BP1 also plays an important role in COAD pathogenesis. Its deletion downregulates k-RAS expression downstream of *β*-catenin and simultaneously inhibits colon cancer cell proliferation, whereas IGF2BP1 overexpression increases c-MYC and K-RAS expression and promotes colon cancer cell proliferation [40]. Whether MIR210HG is involved in this pathway in COAD requires further investigation. In gastric cancer, EIF3J-DT is involved in the regulation of autophagy and chemical resistance of gastric cancer cells by targeting ATG14 [41], while autophagy-dependent apoptosis has been shown to be a promising therapeutic target in COAD [42]. ZEB1-AS1 is involved in the regulation of the ZEB1 pathway; its activation has been reported to promote the stem characteristics and invasiveness of COAD cells [32, 43]. The aforementioned evidence suggests functional mechanisms by which the lncRNAs we identified may be involved in COAD and suggests ways for improving chemotherapy sensitivity and prognosis. Considering our insufficient understanding of these lncRNAs, further studies on them are of clear clinical value.

We found decreased sensitivity to multiple chemotherapeutic agents in the high-risk group stratified by CPR-related prognosis. The development of chemoresistance is an important factor that limits the therapeutic efficacy of anticancer drugs and ultimately leads to the failure of COAD chemotherapy [44]. Transport-based mechanisms of cellular drug resistance play important roles [45, 46]. Through the control of entry and exit of substrates through the cell membrane by membrane transporters, such as P-gp, multiple drugs can escape from cancer cells, decreasing their intracellular accumulation, resulting in multidrug resistance (MDR) that is not limited to a specific type and confers resistance to multiple drugs [47]. Studies on MDR mechanisms and strategies for their reversal play an important role in the success of chemotherapy [48–50]. There have been studies showing that a new class of thiosemicarbazone compounds, the copper-binding di-2-pyridyl ketone thiosemicarbazones, has great promise. Through a unique mechanism, they form redox-active complexes with copper in the lysosomes of cancer cells to reduce the amount of copper in the body, thereby overcoming P-gp-mediated MDR [51]. Therefore, chelators that bind copper have been developed as anticancer agents [51]. Our data on decreased sensitivity to multiple chemotherapeutic agents in patients with COAD in the lncRNA-stratifiedhigh-risk group may also be due to higher Cu concentrations. The targeted application of chelators that bind copper to fight cancer progression and chemoresistance has significant clinical potential.

In conclusion, we identified ten cuproptosis-related lncRNAs using the multivariate Cox regression analysis and constructed a risk model that can accurately predict COAD prognosis. This evidence provides a foundation for future research on COAD. Our study had some limitations. All analyses were performed using a TCGA-COAD cohort and have not been validated against other databases. Additionally, *in vivo* and *in vitro* experiments should be performed for further validation. Further exploration of the impact of cuproptosis on prognosis and chemotherapy resistance in COAD may provide new ideas for further study and clinical applications.

## Data Availability

All data and clinical information involved in this study were obtained from a public database (https://portal.gdc.cancer.gov/) approved by the ethics committee.

## Supplementary Materials

Supplementary Materials Supplementary Table 1: a table includes 870 cuproptosis-related lncRNAs. Supplementary Table 2: a table includes 15 cuproptosis-related lncRNAs associations with COAD OS.
