# Supplementary material for: A Prognostic Cuproptosis-Related LncRNA Signature for Colon Adenocarcinoma
Source: J Oncol. 2023 Feb 17;2023:5925935. doi: 10.1155/2023/5925935 (PMC9957631; doi:10.1155/2023/5925935)
Supplement: Supplementary Materials — Supplementary Table 1: a table includes 870 cuproptosis-related lncRNAs. Supplementary Table 2: a table includes 15 cuproptosis-related lncRNAs associations with COAD OS. [file 5925935.f1.zip › Supplementary Table 1.docx]

| Cuproptosis-related lncRNA |
| --- |
| ABALON |
| AL353804.1 |
| CYTOR |
| MAP3K20-AS1 |
| AC084125.4 |
| AC007406.5 |
| MUC20-OT1 |
| AC093788.1 |
| AL158212.3 |
| AL161729.4 |
| AP006621.2 |
| CCDC18-AS1 |
| AF131215.5 |
| LINC02362 |
| AL356356.1 |
| LINC00467 |
| AC005005.3 |
| AC129510.1 |
| DHRS4-AS1 |
| AC011468.1 |
| LINC01816 |
| AL731571.1 |
| AC015982.1 |
| AC015813.1 |
| AFDN-DT |
| AL034550.1 |
| AC106900.2 |
| HNF4A-AS1 |
| LINC02035 |
| AC010976.2 |
| AC092338.1 |
| AC004918.3 |
| AC018695.4 |
| AC005586.1 |
| LINC-PINT |
| AL133410.1 |
| AL021578.1 |
| AL139089.1 |
| AC110285.6 |
| AL109614.1 |
| AC009120.2 |
| AC069234.4 |
| AC009948.1 |
| LINC00578 |
| AP003469.4 |
| AC080129.2 |
| AL080317.1 |
| AP000240.1 |
| LINC01578 |
| MIR600HG |
| AL022313.2 |
| MNX1-AS1 |
| EIF1AX-AS1 |
| AC021078.1 |
| ZNF710-AS1 |
| AL049539.1 |
| SNHG16 |
| CD44-AS1 |
| AC090181.2 |
| AL133243.2 |
| AL157394.1 |
| DCST1-AS1 |
| AP001330.5 |
| AC009065.2 |
| OCIAD1-AS1 |
| AL163051.1 |
| LINC02595 |
| AC092953.2 |
| AC092747.4 |
| CIRBP-AS1 |
| AL158166.1 |
| CASC19 |
| EP300-AS1 |
| AC013403.2 |
| SCAMP1-AS1 |
| AC124312.5 |
| SNHG1 |
| AL359962.1 |
| AC097376.2 |
| AC009120.3 |
| AC011477.2 |
| DNM3OS |
| AC091057.1 |
| AL049555.1 |
| AL513327.1 |
| AL139289.1 |
| AL606834.1 |
| Z83843.1 |
| AC080013.6 |
| AC145423.2 |
| AC011477.3 |
| AC093157.1 |
| NCBP2-AS1 |
| AC104113.1 |
| AC068580.3 |
| AC016746.1 |
| AC084036.1 |
| AL391834.1 |
| AL133520.1 |
| ALG13-AS1 |
| ATP1A1-AS1 |
| AL355338.1 |
| TRIM31-AS1 |
| AL109811.2 |
| PSPC1-AS2 |
| AL136221.1 |
| AL512413.1 |
| AL136531.1 |
| ITCH-IT1 |
| AC016394.1 |
| AP003774.4 |
| AGAP1-IT1 |
| JPX |
| LINC01811 |
| AC090948.2 |
| AC092868.2 |
| AC137932.1 |
| HCG18 |
| AC068580.1 |
| SNHG4 |
| AL137782.1 |
| AL117381.1 |
| AL139246.5 |
| AC139887.1 |
| AP003352.1 |
| ZNF32-AS2 |
| FAM222A-AS1 |
| AC091563.1 |
| AC087588.2 |
| AC015922.2 |
| AL355353.1 |
| AL133371.2 |
| AC011374.2 |
| ZNF674-AS1 |
| FLJ20021 |
| AC004233.3 |
| AL354696.1 |
| AC090948.1 |
| GK-IT1 |
| TAPT1-AS1 |
| ATP1B3-AS1 |
| AC103760.1 |
| AC010168.2 |
| AC087752.4 |
| ACTA2-AS1 |
| AP001429.1 |
| AC018809.2 |
| LINC01278 |
| AC015922.3 |
| TBILA |
| AC092894.1 |
| AC092279.1 |
| WAC-AS1 |
| HCG11 |
| AC092171.4 |
| ACBD3-AS1 |
| AL513550.1 |
| AC006042.1 |
| AC011451.1 |
| AL035563.1 |
| AC010761.1 |
| AL451042.1 |
| AL121992.3 |
| AC027644.3 |
| FLJ46906 |
| PXN-AS1 |
| CARD8-AS1 |
| AC112496.1 |
| AC004492.1 |
| AC005519.1 |
| CNNM3-DT |
| AC092611.2 |
| AC008124.1 |
| AC132192.2 |
| AC093297.2 |
| CFTR-AS1 |
| AC253536.3 |
| TMEM92-AS1 |
| LINC01748 |
| LINC01876 |
| HOXB-AS2 |
| AC024075.1 |
| AL590723.1 |
| HOTAIRM1 |
| AL121603.2 |
| AL391244.1 |
| AC009318.2 |
| OIP5-AS1 |
| AP000254.1 |
| AC010834.3 |
| GAS5 |
| Z82243.1 |
| PSMA3-AS1 |
| AC037198.1 |
| AL031985.3 |
| SNHG19 |
| AC092794.1 |
| AL359881.1 |
| AC004477.3 |
| AL031670.1 |
| NUTM2A-AS1 |
| AC002064.2 |
| LINC01705 |
| GPRC5D-AS1 |
| AC008537.2 |
| AC092119.2 |
| AC005332.4 |
| AL049840.5 |
| AL136084.3 |
| AC008870.2 |
| AGBL5-IT1 |
| AC004837.2 |
| LINC00339 |
| LINC01224 |
| AC078883.1 |
| MALAT1 |
| AL137003.2 |
| ZNF460-AS1 |
| AL604028.1 |
| AP000873.2 |
| AL592148.3 |
| AC025165.5 |
| AC002116.2 |
| MIR3936HG |
| AL031716.1 |
| NNT-AS1 |
| AC090517.2 |
| AC078778.1 |
| AC074117.1 |
| AL353804.2 |
| AC004943.2 |
| TPM1-AS |
| OSER1-DT |
| AC024075.3 |
| LMO7-AS1 |
| AL138963.1 |
| AL050341.2 |
| AC007255.1 |
| POLH-AS1 |
| AL354993.2 |
| AC002128.2 |
| AC010542.5 |
| AL137779.2 |
| MAPKAPK5-AS1 |
| AL078587.1 |
| AL606489.1 |
| AC010487.2 |
| AC132872.3 |
| AC008280.3 |
| AC009506.1 |
| AL139287.1 |
| AC022211.1 |
| AC090948.3 |
| LINC01637 |
| AC074032.1 |
| AC012073.1 |
| AL117336.2 |
| AC010531.6 |
| AC108727.1 |
| AC022098.1 |
| AC023157.3 |
| AC121761.1 |
| ZBED5-AS1 |
| AC100814.1 |
| AC018809.1 |
| AL590764.1 |
| AC106820.3 |
| AC253536.6 |
| LINC00852 |
| AC060780.1 |
| EPB41L4A-AS1 |
| HIF1A-AS2 |
| AC016831.4 |
| RAD51-AS1 |
| AL080317.2 |
| AL592546.1 |
| AC022973.3 |
| AC020915.2 |
| AL035071.2 |
| AC092168.2 |
| AC138028.4 |
| AL355388.1 |
| AL035661.1 |
| CR936218.1 |
| AC005261.3 |
| AC007878.1 |
| AP001628.1 |
| AL158166.2 |
| ZNF529-AS1 |
| AC092535.4 |
| AC130456.3 |
| USP46-AS1 |
| AC007342.5 |
| AL022328.3 |
| AC090116.1 |
| UBR5-AS1 |
| AC006504.8 |
| AC015849.3 |
| AC048341.2 |
| AC069307.1 |
| AC127024.5 |
| GAS5-AS1 |
| AC019330.1 |
| AL596202.1 |
| AC078860.1 |
| AC004067.1 |
| AC009318.3 |
| AL353796.1 |
| RNASEH1-AS1 |
| ZFAS1 |
| AL031673.1 |
| AL390208.1 |
| AC106876.1 |
| AC137630.3 |
| TRIM52-AS1 |
| AC007608.2 |
| FBXL19-AS1 |
| AC018638.7 |
| PDXDC2P-NPIPB14P |
| FTX |
| AC018521.6 |
| AC091588.1 |
| AL138756.1 |
| AL450384.2 |
| AC009054.2 |
| AL117379.1 |
| DNAJC9-AS1 |
| BX322234.1 |
| AC138207.2 |
| C1RL-AS1 |
| AC019080.5 |
| AC125257.1 |
| SPINT1-AS1 |
| AC080112.1 |
| AC138696.2 |
| TP53TG1 |
| AC027607.1 |
| MIATNB |
| STX17-AS1 |
| AL359715.3 |
| AC008966.1 |
| AC084824.5 |
| AC124016.2 |
| AC008982.2 |
| AP001469.3 |
| AL139393.2 |
| AL031775.1 |
| AP002336.2 |
| THAP9-AS1 |
| CASC9 |
| ITGA6-AS1 |
| AC007485.1 |
| AC009065.4 |
| MEG3 |
| FLNB-AS1 |
| LINC01558 |
| AC025181.2 |
| AC116366.1 |
| WNT5A-AS1 |
| AC079414.3 |
| AC009032.1 |
| AL122035.1 |
| AL355488.1 |
| AL049840.3 |
| LINC00997 |
| RARA-AS1 |
| AC007996.1 |
| SNHG7 |
| MIAT |
| AC015871.3 |
| AC010326.3 |
| UBE2R2-AS1 |
| AC116914.2 |
| AC020913.3 |
| AC018653.3 |
| SNHG11 |
| AC007849.1 |
| AC022306.2 |
| AC008770.3 |
| TRAM2-AS1 |
| AC002550.2 |
| SH3BP5-AS1 |
| AL596325.2 |
| AC110792.3 |
| AC018645.2 |
| AC108673.2 |
| AC010547.2 |
| AL355075.4 |
| EBLN3P |
| AC090617.5 |
| MIR210HG |
| AC139887.2 |
| NPTN-IT1 |
| AC124045.1 |
| AC020663.2 |
| MIR17HG |
| AC124283.2 |
| CARMN |
| GABPB1-AS1 |
| AL133330.1 |
| AC018690.1 |
| SLC12A9-AS1 |
| LINC02562 |
| AP000866.6 |
| PRKCQ-AS1 |
| AL391121.1 |
| GK-AS1 |
| AC006547.1 |
| TMEM161B-AS1 |
| AL451042.2 |
| SDCBP2-AS1 |
| AC025857.2 |
| AL121895.2 |
| AC005261.1 |
| LINC01123 |
| AC005674.2 |
| FAM111A-DT |
| LINC01006 |
| ERVK13-1 |
| AC012615.1 |
| AL512791.1 |
| MIRLET7BHG |
| AC127502.2 |
| AC115618.2 |
| SNHG20 |
| AC006449.2 |
| AC007128.1 |
| AC108449.2 |
| AL157932.1 |
| AC008906.1 |
| SBF2-AS1 |
| AL157786.1 |
| AC120053.1 |
| AC022150.4 |
| AC005034.5 |
| TPT1-AS1 |
| AL162724.2 |
| MACC1-AS1 |
| AL445472.1 |
| APTR |
| GAS6-AS1 |
| AC010761.3 |
| LINC00513 |
| HM13-IT1 |
| AP000692.1 |
| AC127024.4 |
| AC018926.3 |
| AC083843.2 |
| AC073651.1 |
| RGMB-AS1 |
| AC141002.1 |
| AC004908.1 |
| EIF3J-DT |
| AC092902.2 |
| AC008771.1 |
| AC121338.2 |
| HOXA10-AS |
| AC010536.2 |
| AC087276.1 |
| AC138932.5 |
| AC093726.1 |
| AP001318.2 |
| AL138689.1 |
| ADNP-AS1 |
| AL354707.1 |
| FGD5-AS1 |
| ATP2A1-AS1 |
| MIR155HG |
| U62317.2 |
| AC058791.1 |
| AL049869.3 |
| MKLN1-AS |
| AC124067.4 |
| AC091057.4 |
| AC093110.1 |
| AL357033.3 |
| LINC02441 |
| AC116345.1 |
| AC012181.2 |
| AC025287.3 |
| AC132807.2 |
| AC084824.4 |
| AC124067.2 |
| AC000123.1 |
| AC005332.6 |
| AC093227.1 |
| AC012317.1 |
| HNF1A-AS1 |
| AC010260.1 |
| AL163051.2 |
| LINC01138 |
| LINC00654 |
| AL035071.1 |
| AL442125.2 |
| Z69706.1 |
| AC005253.1 |
| AC004066.2 |
| AL442067.1 |
| AC004908.2 |
| AC096921.2 |
| PLBD1-AS1 |
| GARS-DT |
| TMEM9B-AS1 |
| AC087222.1 |
| LINC01184 |
| AP000786.1 |
| AC145207.8 |
| LINC02577 |
| AC008669.1 |
| RUSC1-AS1 |
| ZKSCAN2-DT |
| AC005046.1 |
| AC040169.1 |
| HOXB-AS4 |
| AL445222.1 |
| NEAT1 |
| MAFG-DT |
| AC025580.2 |
| AC073487.1 |
| AC245297.3 |
| FMR1-IT1 |
| SP2-AS1 |
| AL359076.1 |
| AL031275.1 |
| CHKB-DT |
| AL133406.2 |
| AC108058.1 |
| AC068790.5 |
| MCF2L-AS1 |
| DNMBP-AS1 |
| AC008649.2 |
| AC009812.4 |
| AC005083.1 |
| AC093732.1 |
| ASH1L-AS1 |
| OTUD6B-AS1 |
| AC020978.3 |
| AC036214.2 |
| AL021707.6 |
| AC108134.3 |
| LINC01186 |
| LINC00342 |
| AC099518.2 |
| IQCH-AS1 |
| AC132872.1 |
| AC026368.1 |
| NUTM2B-AS1 |
| AC097461.1 |
| AL160006.1 |
| AC145098.1 |
| HLA-DQB1-AS1 |
| AC104699.1 |
| AC245014.3 |
| LINC02381 |
| AL136295.7 |
| AC005104.1 |
| AC010973.2 |
| THUMPD3-AS1 |
| AL117382.1 |
| AL391834.2 |
| AC093799.1 |
| AL078581.1 |
| AC092171.2 |
| AC097639.1 |
| BACE1-AS |
| GASAL1 |
| AL158837.1 |
| DLEU2 |
| AC004253.1 |
| LINC02001 |
| AL121832.3 |
| TOLLIP-AS1 |
| AP002387.1 |
| AL132989.1 |
| AC100830.2 |
| AC004656.1 |
| AC024060.1 |
| AL022323.1 |
| RAB11B-AS1 |
| AC108010.1 |
| AC079684.1 |
| LINC01176 |
| AL031775.2 |
| LINC02163 |
| NIFK-AS1 |
| SREBF2-AS1 |
| AC087277.2 |
| NRSN2-AS1 |
| AC090425.1 |
| AC022400.1 |
| DGUOK-AS1 |
| AC048344.4 |
| AC123023.1 |
| AC068888.1 |
| AC115618.1 |
| AC022762.2 |
| AP005482.3 |
| Z68871.1 |
| AL137003.1 |
| AC005332.3 |
| AL365226.2 |
| AC097641.2 |
| VPS9D1-AS1 |
| AL022322.1 |
| AC023043.4 |
| MCM3AP-AS1 |
| AC012467.1 |
| AL022328.1 |
| FOXD2-AS1 |
| FIRRE |
| AC073335.2 |
| AC010719.1 |
| AL117336.3 |
| ANKRD10-IT1 |
| AC068152.1 |
| AC096992.2 |
| LINC00265 |
| NDUFV2-AS1 |
| AL021707.3 |
| AC007637.1 |
| AL021707.8 |
| SYNPR-AS1 |
| LINC01003 |
| AF129075.1 |
| TMPO-AS1 |
| AC000061.1 |
| AC004264.1 |
| AC096947.1 |
| AC107952.2 |
| AL034397.3 |
| AC232271.1 |
| AC008915.2 |
| AC002553.2 |
| ARHGEF38-IT1 |
| PLAC4 |
| AP002840.2 |
| RAB30-AS1 |
| AC022211.3 |
| AL024508.1 |
| ACAP2-IT1 |
| MIR100HG |
| USP12-AS1 |
| AC104695.3 |
| AC090739.1 |
| AL360181.2 |
| AC007383.2 |
| ZDHHC20-IT1 |
| MID1IP1-AS1 |
| DNAJC3-DT |
| AP003392.1 |
| Z98884.2 |
| MIR222HG |
| AC004241.3 |
| AC008764.2 |
| AC069234.5 |
| AC007991.2 |
| PAXIP1-AS2 |
| AL033384.2 |
| AC019131.2 |
| AL122010.1 |
| MIR4435-2HG |
| AC138207.4 |
| INE1 |
| AC138956.1 |
| MIR181A2HG |
| AC005034.3 |
| MHENCR |
| LINC01534 |
| LINC01614 |
| AC011476.3 |
| AC012557.1 |
| PVT1 |
| AC018752.1 |
| AL359915.2 |
| FOXN3-AS1 |
| RMRP |
| AC135050.5 |
| PCCA-AS1 |
| AC008035.1 |
| AC099850.3 |
| AF117829.1 |
| AC008760.1 |
| PAN3-AS1 |
| AL139011.1 |
| AL354733.3 |
| LINC01106 |
| AC005899.6 |
| AC004918.1 |
| LINC00909 |
| MBNL1-AS1 |
| AC073046.1 |
| AC002401.3 |
| LINC02298 |
| LINC01473 |
| LINC01315 |
| AL928654.2 |
| AC008750.4 |
| SMIM25 |
| AC018797.2 |
| LINC00294 |
| SNHG9 |
| AL158801.3 |
| AC008115.3 |
| TNFRSF10A-AS1 |
| AL121894.2 |
| AC025171.1 |
| AC012170.2 |
| AL049840.4 |
| AC099343.2 |
| CH17-340M24.3 |
| AC009237.15 |
| AC007566.1 |
| AL121832.2 |
| AL161891.1 |
| AC004148.2 |
| YTHDF3-AS1 |
| AC012640.2 |
| AL136115.2 |
| AL391001.1 |
| BX537318.1 |
| LINC01355 |
| AC124319.1 |
| AP001453.2 |
| RPARP-AS1 |
| AP001033.2 |
| AC093484.4 |
| AC067838.1 |
| AL136320.1 |
| AC127521.1 |
| N4BP2L2-IT2 |
| AL139407.1 |
| AP002360.3 |
| AC009118.3 |
| AP002907.1 |
| AC234772.2 |
| ZEB1-AS1 |
| AL163953.1 |
| AC063965.1 |
| AL049840.2 |
| AC069281.2 |
| AL359715.1 |
| AL021878.2 |
| AC073569.2 |
| DGCR11 |
| AC010186.3 |
| AC087481.3 |
| AL606834.2 |
| TMEM44-AS1 |
| AL731566.1 |
| AL392172.1 |
| AL731567.1 |
| AL450263.1 |
| AP003486.1 |
| SNHG8 |
| MMP25-AS1 |
| AL354920.1 |
| AL049552.1 |
| AP003068.1 |
| AC062037.2 |
| SNHG6 |
| AL117382.2 |
| AP005899.1 |
| AC098484.1 |
| AC012181.1 |
| AP001486.2 |
| AL117332.1 |
| AC096586.2 |
| LINC01004 |
| AC080013.4 |
| AC073957.3 |
| URB1-AS1 |
| AC073896.3 |
| AL121839.2 |
| FAM160A1-DT |
| SNHG22 |
| AC131971.1 |
| ZNF433-AS1 |
| MAGI2-AS3 |
| AC109460.2 |
| LINC00641 |
| AL157392.3 |
| EXOC3-AS1 |
| TSPOAP1-AS1 |
| AC147651.3 |
| AC004593.1 |
| AC092755.1 |
| FP671120.4 |
| AC243967.2 |
| AC055822.1 |
| AC017074.1 |
| AC011676.1 |
| XIST |
| AL355075.2 |
| AC027097.1 |
| LINC01560 |
| AC080162.1 |
| AC138207.5 |
| AC022509.3 |
| AL391863.2 |
| LENG8-AS1 |
| PPP3CB-AS1 |
| IPO5P1 |
| AP001625.2 |
| C5orf56 |
| AL359513.1 |
| PRR7-AS1 |
| AC026124.2 |
| AL139384.1 |
| AC016727.1 |
| AC002553.1 |
| LINC01806 |
| HOXA-AS2 |
| AC113143.1 |
| AL157838.1 |
| AGAP2-AS1 |
| AC090589.3 |
| AL139120.1 |
| AC009041.4 |
| AC009113.1 |
| AC004130.1 |
| AL121772.3 |
| AL355001.2 |
| NORAD |
| AL365277.1 |
| AC090198.1 |
| LINC01023 |
| AC007938.3 |
| SCARNA9 |
| AC116407.2 |
| DLEU1 |
| LINC01235 |
| AC090579.1 |
| AC245884.8 |
| AC084117.1 |
| AC009269.5 |
| SNHG3 |
| ZNF236-DT |
| SOS1-IT1 |
| AC104695.2 |
| AC104170.1 |
| NCK1-DT |
| AC020915.3 |
| AC138956.2 |
| AC002128.1 |
| AC107068.1 |
| AC079907.1 |
| AC139795.2 |
| CEBPA-DT |
| AL356299.2 |
| AC107027.3 |
| AL357079.1 |
| AC245100.7 |
| AC007038.2 |
| AC012467.2 |
| ARMCX5-GPRASP2 |
| AC103591.3 |
| PCED1B-AS1 |
| LINC02487 |
| AC026356.1 |
| AC098851.1 |
| AC083799.1 |
| AC104083.1 |
| AC009812.1 |
| AC083880.1 |
| NFYC-AS1 |
| AL390728.6 |
| AC006333.2 |
| AC087286.2 |
| AC135050.6 |
| AL049840.1 |
