# Supplementary material for: A Prognostic Cuproptosis-Related LncRNA Signature for Colon Adenocarcinoma
Source: J Oncol. 2023 Feb 17;2023:5925935. doi: 10.1155/2023/5925935 (PMC9957631; doi:10.1155/2023/5925935)
Supplement: Supplementary Materials — Supplementary Table 1: a table includes 870 cuproptosis-related lncRNAs. Supplementary Table 2: a table includes 15 cuproptosis-related lncRNAs associations with COAD OS. [file 5925935.f1.zip › Supplementary Table 2.docx]

| AL161729.4 |
| --- |
| AC068580.3 |
| AL138756.1 |
| MIR210HG |
| AC007128.1 |
| EIF3J-DT |
| LINC02381 |
| AC010973.2 |
| AC008760.1 |
| TNFRSF10A-AS1 |
| AC004148.2 |
| RPARP-AS1 |
| ZEB1-AS1 |
| AC087481.3 |
| AC073957.3 |
